# Individual differences in metabolomics: individualised responses and between-metabolite relationships

**DOI:** 10.1007/s11306-012-0414-8

**Published:** 2012-03-15

**Authors:** Jeroen J. Jansen, Ewa Szymańska, Huub C. J. Hoefsloot, Age K. Smilde

**Affiliations:** 1 Netherlands Metabolomics Centre, Einsteinweg 55, 2333 CC Leiden, The Netherlands; 2 Biosystems Data Analysis Group, Swammerdam Institute for Life Sciences, Faculty of Sciences, Universiteit van Amsterdam, Science Park 904, 1098 XH Amsterdam, The Netherlands; 3 Department of Analytical Chemistry, Institute for Molecules and Materials, Radboud University Nijmegen, Toernooiveld 1, 6525 ED Nijmegen, The Netherlands

**Keywords:** Individual difference, INDSCAL, Glucosinolate, Induced response, Herbivory, Chemometrics, Multivariate data analysis, PARAFAC, Simultaneous component analysis

## Abstract

Many metabolomics studies aim to find ‘biomarkers’: sets of molecules that are consistently elevated or decreased upon experimental manipulation. Biological effects, however, often manifest themselves along a continuum of individual differences between the biological replicates in the experiment. Such differences are overlooked or even diminished by methods in standard use for metabolomics, although they may contain a wealth of information on the experiment. Properly understanding individual differences is crucial for generating knowledge in fields like personalised medicine, evolution and ecology. We propose to use simultaneous component analysis with individual differences constraints (SCA-IND), a data analysis method from psychology that focuses on these differences. This method constructs axes along the natural biochemical differences between biological replicates, comparable to principal components. The model may shed light on changes in the individual differences between experimental groups, but also on whether these differences correspond to, e.g., responders and non-responders or to distinct chemotypes. Moreover, SCA-IND reveals the individuals that respond most to a manipulation and are best suited for further experimentation. The method is illustrated by the analysis of individual differences in the metabolic response of cabbage plants to herbivory. The model reveals individual differences in the response to shoot herbivory, where two ‘response chemotypes’ may be identified. In the response to root herbivory the model shows that individual plants differ strongly in response dynamics. Thereby SCA-IND provides a hitherto unavailable view on the chemical diversity of the induced plant response, that greatly increases understanding of the system.

**Electronic supplementary material:**

The online version of this article (doi:10.1007/s11306-012-0414-8) contains supplementary material, which is available to authorized users.

## Introduction

Ronald Fisher, in his landmark paper introducing Analysis of Variance (ANOVA), already stated that although mendelian genetic variation is discrete, it may lead to continuous phenotypic differences between replicates (Fisher 1918). Such individual phenotypic differences may be key to biological success and survival (Steppan et al. 2002), because individuals with a specifically fine-tuned response that leads to higher fitness are favoured over their peers. Individual differences are therefore the main driving force for evolutionary change (Dall et al. 2004).

However, in life sciences (from agricultural to medical research) the main goal is to find responses that are reproducible between most individuals. This stems from the main objectives in these fields, i.e., providing consistently high crop yields or curing as many people as possible with a given treatment. This focus on reproducibility resonates into the statistical methods of choice: the heirs of Ronald Fisher at Rothamsted Research Centre 100 years later still quantify differences in plant phenotypes caused by bacterial infection (Ward et al. 2010) with his ANOVA method (Sokal and Rohlf 1995), although now with state-of-the-art metabolomics technology.

Biological systems have become observable in much more detail than in the time of Fisher. The full complement of genes and a large number of proteins and metabolites can be measured by ‘omics’ platforms, which in turn become more and more high-throughput such that ever larger numbers of individuals can be characterised. This broadened view has induced modern systems biology to embrace another biological principle into data analysis, namely that all these genes, proteins and metabolites are interrelated through biochemical pathways. Multivariate methods such as partial least squares-discriminant analysis (PLS-DA) and principal component analysis (PCA) (Jansen et al. 2009a, b; Trygg et al. 2007; Lindon et al. 2000) reflect these relations much better than ANOVA. However, still the consistent differences induced by a treatment are sought by these methods, such that patterns of individual differences are lost.

The conceptual models behind many such individual metabolic differences dictate that these should have a specific structure. For example, plants generally have a limited amount of energy at their disposal, which they will have to distribute among several processes upon herbivore attack. Most importantly, they need to choose between defending themselves chemically or to compensate the resulting damage by growth (Herms and Mattson 1992), which will induce a negative relationship between both mechanisms. These relations will also be visible in the biochemical profiles of mutually associated metabolites, proteins and transcripts associated with the respective pathways. The resulting structured variation is beyond reach of most ‘standard’ multivariate statistical methods, but may be described by a dedicated data analysis method.

Currently, no component analysis method is available that both focuses on individual differences, while describing the specific response of each individual biological replicate. The individual differences scaling (INDSCAL) (Carroll and Chang 1970; Harshman and Lundy 1984) method—recently proposed for metabolomics (Jansen et al. 2011)—describes ‘between metabolite relationships’ (BMRs) that are closely related to the individual biochemical differences between biological replicates. However, INDSCAL describes these differences on the level of the experimental groups but does not reach the level of the individual biological replicate. On the other hand, simultaneous component analysis (SCA) (Ten Berge et al. 1992; Timmerman and Kiers 2003) may be used to identify the distribution of the individuals within an experimental group, which may lead to a priori unknown clusters belonging to, e.g., non-responders to the experimental manipulation. However, the results of SCA ‘with equal profiles’ (SCA-P) (Jansen et al. 2005; Smilde et al. 2005b; Jansen et al. 2004) do not give a straightforward interpretation of the group-level differences. Because INDSCAL and SCA-P are different methods, the individual and group-levels do not commute between both models.

In this manuscript we propose a method to analyse and interpret individual differences on the individual and group-level simultaneously. This method is called SCA-IND and mixes the specific constraints from INDSCAL with the SCA model, such that entire experimental groups and individual biological replicates can be analysed simultaneously. Subsequently, we discuss whether covariances or correlations better reflect the aspects of BMRs that are most appropriate for the individual differences, which is directly used in SCA-IND. Finally the SCA-IND model is applied to reveal the intricacies of the chemical response of cabbage plants to herbivory. The relations between metabolites, tied tightly together with individual differences metabolomics, have been proposed before as a very appropriate perspective to observe induced responses to biotic and abiotic plant stress (Broeckling et al. 2005).

## Theory

### Different levels of individual biochemical differences

Metabolomic data consists of comprehensive biochemical characterization of biological samples, often as levels of previously identified metabolites, present in a database (i.e., metabolic profiling) (Dunn and Ellis 2005). In metabolomics studies experimental factors (such as doses of a toxicant or the origin of a population) are manipulated and the resulting metabolic change is then measured, generally for multiple biological replicates. The subdivision of metabolic variation with respect to experimental groups and natural variation between biological replicates is given in Eq. 1.

1 $$ {\mathbf{X}}_{k} = 1_{{I_{k} }} {{\upmu}}^{\text{T}} + 1_{{I_{k} }} {{\upalpha}}_{k}^{\text{T}} + {\mathbf{B}}_{k} $$

where **X**_*k*_ is the (*I*_*k*_ × *J*) matrix of measured levels of metabolites 1…*j* …*J* for biological replicates 1_*k*_ …*i*_*k*_ …*I*_*k*_ of experimental group *k*, μ is the length *J* vector containing the average metabolite levels of all replicates in all experimental groups, vector α_*k*_ contains the average metabolite levels for all biological replicates of group *k* and α_*k*_ is expressed as a deviation from μ, leading to $\sum\nolimits_{k = 1}^{K} {I_{k} {{\upalpha}}_{k}^{\text{T}} } = 0^{\text{T}}$; matrix** B**_*k*_ contains the deviation of each individual biological replicate from α_*k*_, such that $1_{{I_{k} }}^{\text{T}} {\mathbf{B}}_{k} = 0$.

Equation 1 is central to most data analysis techniques in current use for metabolomics: it defines a contribution equal for all individuals μ and disentangles the remaining metabolic variation in matrix **X**_*k*_ into a contribution α_*k*_ equal for all individuals in one experimental group and a contribution **B**_*k*_ specific for each individual within each group. For complex experimental designs, *k* can be built up from contributions by different factors [see e.g. (Smilde et al. 2005a)]. In most metabolomics studies, interest lies in characterizing and statistically assessing the differences between different group means, i.e. between α_*k*_. The individual differences in **B**_*k*_ are then treated as a nuisance. Their contribution is either regarded in the light of clustering individuals according to α_*k*_, e.g., by PCA and ANOVA-SCA (Zwanenburg et al. 2010) or minimized to describe the differences between α_*k*_, e.g., by the Fisher ratio (Smit et al. 2008) in PLS-DA models.

The individual differences in **B**_*k*_ may contain three types of insightful information that are of interest in metabolomics studies. These three types of information refer to three levels of variation related to

1. The individuals with the most pronounced response to an experimental manipulation. That could be used to select them for follow-up experiments.
2. The distribution of the response magnitude within the experimental group. That could be used to distinguish between a subdivision in responders or non-responders or an axis of intensity between the responses of different individuals.
3. A comparison between different experimental groups. That could be used to show that the individual differences within a group of treated individuals are different from a comparable control group.

A single data analysis method should capture these three levels of variation simultaneously, such that the levels can be compared. SCA may be this method.

### Simultaneous component analysis

SCA (Millsap and Meredith 1988; Ten Berge et al. 1992; Timmerman and Kiers 2003; Kiers and Ten Berge 1994) is the model of choice to describe the variation between biological replicates. This model fits the natural variation in all groups (i.e., matrices **B**_*k*_) simultaneously, using component variables familiar from PCA. This allows comparison of the individual differences between groups. The model is given in Eq. 2.

2 $$ \begin{array}{*{20}c} {\text{Model}} & {{\mathbf{B}}_{k}^{{}} = {\mathbf{T}}_{k} {\mathbf{P}_{{}}^{\text{T}}} + {\mathbf{E}}_{k} } \\ {\text{Minimize}} & {f\left( {{\mathbf{T}}_{k} ,\left. {\mathbf{P}} \right|{\mathbf{B}}_{k}^{{}} } \right) = \sum\limits_{k = 1}^{K} {\left\| {{\mathbf{B}}_{k}^{{}} - {\mathbf{T}}_{k} {\mathbf{P}}_{{}}^{\text{T}} } \right\|^{2} } } \\ {\text{subject to}} & { 1^{\textbf{{T}}} {\mathbf{T}}_{k} = 0^{\textbf{{T}}} \forall k} \\ \end{array} $$

where **T**_*k*_ is the (*I*_*k*_ × *R*) matrix containing the SCA scores of group *k*, **P** is the (*J* × *R*) matrix of loadings and *R* is the number of components chosen for the SCA model; **E**_*k*_ is the (*I*_*k*_ × *J*) matrix of model residuals.

The SCA scores (contrary to those of a PCA model on all **X**_*k*_) explicitly describe the individual differences between all individuals within the same experimental group *k* in the scores **T**_*k*_. The metabolites that exhibit many individual differences obtain a large loading value in matrix **P** and the relations between different important metabolites can be interpreted from the signs of the loadings on the same SCA component. This makes the interpretation of the model analogous to PCA—of which SCA is a generalization—and other component models. Individuals with extreme score values of **T**_*k*_ on a specific component can be identified as deviating from the other individuals within the group. Also the distribution of the individual-level scores **T**_*k*_ along the fitted component can be established for each group *k*, to distinguish whether the individual differences within a group can be associated with responders and non-responders—which would lead to score clusters—from a continuous range of individual differences. However, the variation in individual differences between experimental groups, i.e., the third level of **B**_*k*_ is not directly observable from the scores **T**_*k*_. To compare variation in individual differences between experimental groups the individual-level scores **T**_*k*_ need to be translated into group-wide descriptors. The magnitude of the individual differences associated with each component can be calculated by their inner product ${\mathbf{T}}_{k}^{\text{T}} {\mathbf{T}}_{k}$.

The diagonal elements of this (*R* × *R*) symmetric product matrix ${\mathbf{T}}_{k}^{\text{T}} {\mathbf{T}}_{k}$ describe the relative importance of every SCA component *r* in the individual differences of group *k*. However, the different components in matrices **T**_*k*_ interact; the $\frac{1}{2}R\left( {R - 1} \right)$ off-diagonal elements of ${\mathbf{T}}_{k}^{\text{T}} {\mathbf{T}}_{k}$ quantify the magnitude of this interaction. This hampers model interpretation, because also combinations of the components need to be taken into account. This is analogous to the poorer interpretability of Tucker3 compared to that of PARAFAC models (Smilde et al. 2004; Dyrby et al. 2005). This makes the most general variant of SCA—‘SCA with equal profiles’ (SCA-P) which is a PCA model fitted simultaneously on all matrices **B**_*k*_—unfit for this interpretation.

This poor interpretation can be alleviated by imposing additional constraints on ${\mathbf{T}}_{k}^{\text{T}} {\mathbf{T}}_{k}$, usually at the expense of model fit. One such constraint is given in Eq. 3.

3 $$ {\mathbf{T}}_{k}^{\text{T}} {\mathbf{T}}_{k} = {\mathbf{D}}_{k} \forall k,\quad d_{kr} \ge 0\forall k,r $$

where **D**_*k*_ are (*R* × *R*) matrices with nonnegative diagonal elements *d*_*kr*_ and other elements are equal to 0.

This constraint is familiar from the INDSCAL method (Jansen et al. 2011; Carroll and Chang 1970) and allows interpretation of the biochemistry in each component individually. The diagonal values of **D**_*k*_ have the same interpretation as the INDSCAL scores. They are ‘group-level scores’, that show how much variation associated to the BMRs in loadings P is present in every group *k*. However, the INDSCAL method presented before does not give any individual-level scores **T**_*k*_ that underlie **D**_*k*_. The implementation of this constraint in SCA has been described before and is called SCA-IND (Timmerman and Kiers 2003).

The SCA-IND model provides insight on all three levels of information in **B**_*k*_:

1. The individuals are characterized by scores **T**_*k*_: extremely high or low scores indicate individuals that differ much from the average in group *k*. The biochemistry of these differences are given in loadings **P**.

2. By comparing all **T**_*k*_ within an experimental group, the number of individuals differing from the average and the range of these differences among all individuals can be determined.

3. Whether the amount of individual differences changes upon experimental manipulation can be interpreted from the scores **D**_*k*_ between groups *k*. The relations between which metabolites are important in these individual differences can be obtained from the matrix product ${\mathbf{p}}_{r} {\mathbf{p}}_{r}^{\text{T}}$, where **p**_*r*_ is the column of **P** corresponding to the *r*th component.

Although both INDSCAL and SCA-IND provide group-level information through **D**_*k*_, both methods generally do not provide identical results. Both use the same constraint in Eq. 3, yet their minimization criteria differ. The SCA method minimizes $f\left( {{\mathbf{T}}_{k} ,\left. {\mathbf{P}} \right|{\mathbf{B}}_{k}^{{}} } \right)$ in Eq. 2, while INDSCAL minimizes the ‘indirect’ criterion $g\left( {{\mathbf{P}} ,\left. {{\mathbf{D}}_{k} } \right|{\mathbf{B}}_{k}^{{}} } \right) = \left\| {I_{k}^{ - 1} {\mathbf{B}}_{k}^{\text{T}} {\mathbf{B}}_{k}^{{}} - {\mathbf{PD}}_{k} {\mathbf{P}}_{{}}^{\text{T}} } \right\|_{{}}^{2}$, such that both models give different **P** and **D**_*k*_. Both methods are expected to give highly similar results for data without outliers, but $g\left( {{\mathbf{P}} ,\left. {{\mathbf{D}}_{k} } \right|{\mathbf{B}}_{k}^{{}} } \right)$ will give INDSCAL more bias towards individuals that differ considerably from the group average than SCA-IND, because it minimizes the sum-of-squares of the raw data values squared. Furthermore, we cannot think of any pressing biological or chemical grounds to prefer the interpretation of either $g\left( {{\mathbf{P}} ,\left. {{\mathbf{D}}_{k} } \right|{\mathbf{B}}_{k}^{{}} } \right)$ or $f\left( {{\mathbf{T}}_{k} ,\left. {\mathbf{P}} \right|{\mathbf{B}}_{k}^{{}} } \right)$. Therefore, the broader view on the individual differences provided by SCA-IND may be preferred above INDSCAL in most cases. This technical difference does not pose a limit for most operations described before for INDSCAL: the number of components can also be determined by fitting models with increasing numbers of components and comparing the cumulative variation fitted by the model fit_*r*_, using Eq. 4 (Timmerman and Kiers 2003).

4 $$ {\text{fit}}_{r} = \left( {1 - \frac{{\sum\limits_{k = 1}^{K} {\left\| {{\mathbf{B}}_{k}^{{}} - {\mathbf{T}}_{k} {\mathbf{P}}_{{}}^{\text{T}} } \right\|^{2} } }}{{\sum\limits_{k = 1}^{K} {\left\| {{\mathbf{B}}_{k}^{{}} } \right\|^{2} } }}} \right) $$

Also the jack-knife approach described before can be applied in SCA-IND to quantify the confidence in observed group-level differences, given that enough samples are available in the group (Jansen et al. 2011).

The results of the SCA-IND analysis presented in this manuscript have been obtained by algorithms in a package for MATLAB (Mathworks, Natick, MA), which are available for download on http://www.bdagroup.nl/content/Downloads/software/software.php.

### BMRs and individual differences

The BMRs focused upon by INDSCAL are also very relevant to SCA-IND, through the group level of individual differences described by **D**_*k*_. Most literature uses the scalar product matrix ${\mathbf{B}}_{k}^{\text{T}} {\mathbf{B}}_{k}$ to describe BMRs for simplicity of notation, but because the ‘sample variance–covariance matrix’ ${\mathbf{S}}_{k}^{{}} = I_{k}^{ - 1} {\mathbf{B}}_{k}^{\text{T}} {\mathbf{B}}_{k}^{{}}$ can handle unequal numbers of biological replicates per group *k*, this is much wider applicable. Many studies that employ ‘correlation networks’ (see e.g. (Steuer et al. 2003; Weckwerth et al. 2004)) study correlation matrices that express the tightness and linearity of the BMRs. Covariances and correlations are closely related, as Eq. 5 shows.

5 $$ {\mathbf{S}}_{k}^{{}} = {\mathbf{V}}_{k}^{{}} {\mathbf{R}}_{k} {\mathbf{V}}_{k}^{{}} $$

where **S**_*k*_ is the (*J* × *J*) matrix of covariances between the metabolites, **R**_*k*_ is the (*J* × *J*) matrix of Pearson correlations between metabolite descriptors and **V**_*k*_ is the (*J* × *J*) diagonal matrix containing the standard deviations of each metabolite in group *k*.

Equation 5 shows that the covariance is a compound measure that encapsulates the tightness of the relation between two metabolites from the correlation matrix with the magnitude of the individual differences in the levels of these metabolites in matrices **V**_*k*_. Covariances are therefore most relevant to quantify individual biochemical differences and of specific interest to implementation in SCA-IND.

**Fig. 1:**
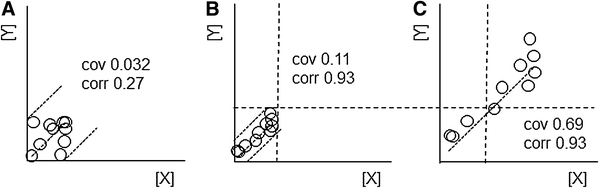
Individual differences: a relationship between metabolites X and Y can emerge, such as the transition from panel **a**–**b**: the individual differences in both individual metabolites are equally large in both panels, but the relation is tighter in panel **b**. An alternative transition would be that both metabolites vary more, while preserving their relation as depicted between panels **b** and **c**. Both the Pearson correlation (CORR) and the covariance (COV) increase for the first transition, but the second transition is only reflected in the covariance

Individual differences between the biological replicates may change in several ways upon experimental manipulation. First of all, experimental manipulations may cause a relation between two metabolites to emerge or disappear, as indicated in the transition from panel a to b in Fig. 1. The individual differences in both metabolites are just as large in panel b as they are in a. In b the two are, however, clearly related, but not in panel a. An appearance of a BMR is reflected in both the correlation and the covariance, as indicated in the corresponding coefficients.

However, many experimental manipulations of interest in systems biology may cause relatively ‘soft’ changes: dietary or lifestyle changes are expected to mostly affect systemic pathways that are involved in the basic functions of the organism. Such pathways are always active and therefore relationships between metabolites may also exist in unperturbed control individuals. A second aspect of such a soft manipulation is that it may affect each individual to a different degree. For example, each individual plant will alter the balance between growth and defence differently upon meeting herbivory. Therefore, the response to soft manipulations may consist of a mutual level increase of several metabolites for all individuals, but the intensity of this increase may be different for each individual. Such a transition is illustrated from Fig. 1b to c, where the relationship between both metabolites is conserved, but the individual differences are much larger in panel c. This transition is also represented well by covariances. Note that the correlation coefficient between the levels of both metabolites does not change, so that such changes cannot be observed by this measure.

By covariances it is possible to pick up the BMR-related variation patterns that are most relevant to individual differences, which makes them preferable to correlation coefficients. A well-known drawback of the covariance is its bias towards metabolites with large concentration variations. However, this aspect transcends individual differences and is relevant to all data analysis methods: large differences in the variation of different metabolites are generally ameliorated by autoscaling, which incidentally corresponds to changing focus from the covariance between metabolites to their correlation. Disregarding this latter aspect, we prescribe the analysis of mean-centered, unscaled data in the search for individual metabolic differences, corresponding to modeling the covariances between metabolites.

### Plant data set

Cabbage plants (*Brassica oleracea*) produce glucosinolates when subjected to herbivory (Bodnaryk 1994). These compounds play a complex ecological role in the plant defence against insect herbivores (Hopkins et al. 2009) and are also of great interest to human health (Fahey et al. 1997). The study compared the effect that herbivory to the shoot (*SJA*) or to the root (*RJA*) has on glucosinolate composition, with that of control plants that did not receive any herbivory. The herbivory was simulated by the application of the hormone jasmonic acid (Bodnaryk 1994). A glucosinolate profiling platform was used to measure the glucosinolate concentrations at 1, 7 and 14 days after the simulated attacks: 11 different glucosinolate species were identified in the plants. This study was described in detail in two earlier papers (Jansen et al. 2011; Jansen et al. 2009a, b): experimental and chemical analysis details about the experiment can be found in the latter reference. Supplementary Table 1 gives the number of biological replicates in every experimental group.

## Results and discussion

**Fig. 2:**
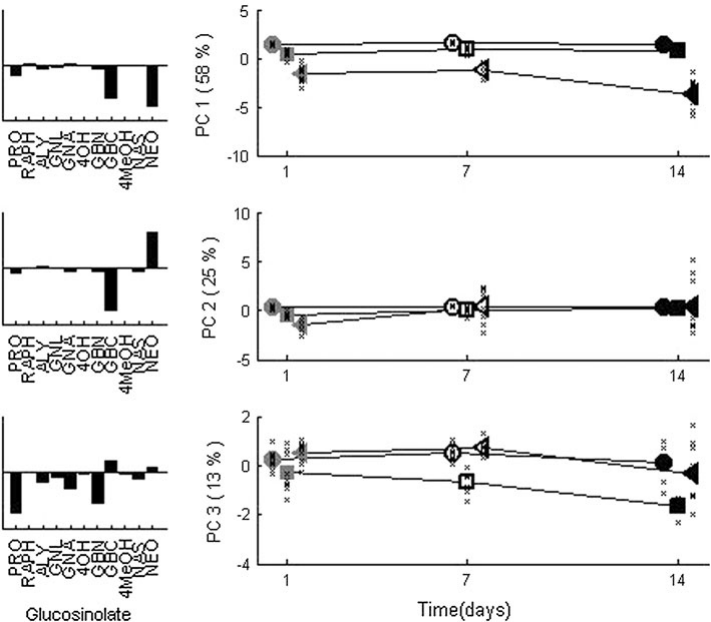
PCA model of glucosinolate level changes: average scores are given for *RJA* upon root induction (*RJA*, *squares*) and shoot induction (*SJA*, *triangles*), together with the control plants (*crosses*). The first component shows a large increase for *SJA* plants and a smaller, significant increase for *RJA* plants, in glucosinolates NEO and GBC; the second component shows a negative relation between the same glucosinolates that is unique to *SJA*. The third component shows an increase in mainly PRO and GBN unique to *RJA*. The crosses for each experimental group indicate the individual plant scores

Both shoot herbivory (*SJA*) and root herbivory (*RJA*) greatly affect plant metabolism, which was already shown before in several PCA-based analyses (van Dam et al. 2010; Jansen et al. 2011; Jansen et al. 2009a, b) and has been repeated in Fig. 2. The response to *SJA* consists of higher Glucobrassicin (GBC) and Neoglucobrassicin (NEO) levels throughout the experiment, where the levels of both glucosinolates become negatively correlated before day 7. Plants that received *RJA* also have higher levels of NEO and GBC, although significantly lower than after *SJA* and without the negative relation between the two. In addition, Progoitrin (PRO) and Glucobrassicanapin (GBN) levels increase between 1 and 7 days after *RJA*.

### Shoot induction

**Fig. 3:**
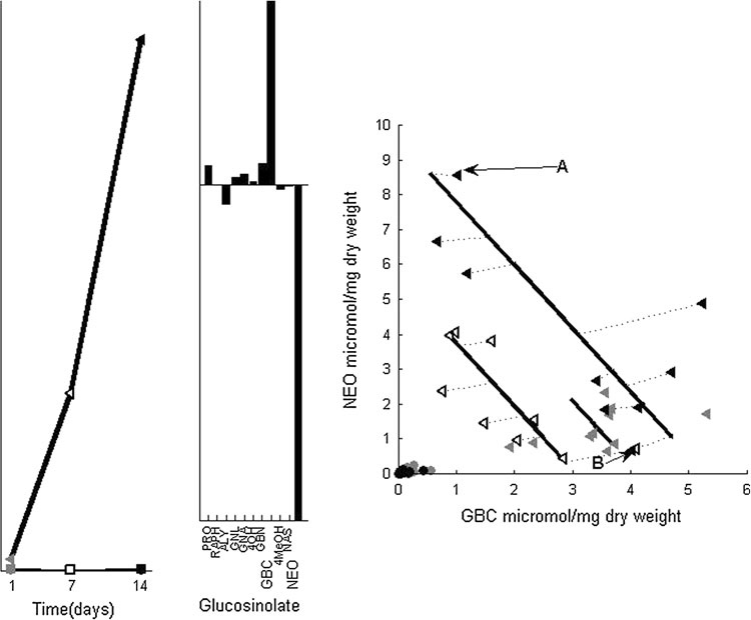
SCA-IND component 1 describes the negative relation between NEO and GBC. *Left* group-level scores, where circles indicate control plants, squares the *RJA* plants and triangles the *SJA* plants *grey labels* show plants harvested after 1 day, white labels after 7 days and *black labels* show plants harvested after 14 days; the *bold time* trajectory belongs to *SJA* plants. *Center* SCA-IND loadings for component 1 that show the negative relation between NEO and GBC. *Right* Measured NEO and GBC levels for the *SJA* plants harvested at all 3 days (indicated by the *symbols*, see leftmost panel for legend). The *lines* indicate the SCA-IND loadings for this component per day, specifically for NEO and GBC. The length of each line shows the range of the individual-level scores for that day, which relate to the group-level scores in the leftmost panel. The *dotted lines* indicate the distance between the measured NEO and GBC levels in plants harvested 7 and 14 days after *SJA* and the prediction of these levels by the SCA-IND model that lie on the *continuous lines*. Along the line belonging to 14 days, plant **a** is indicated as a NEO responder and plant **b** as GBC responder. Note that to make the model results comparable to the measured data, they had to be adjusted with the experimental group-specific values α_*k*_ for NEO and GBC

**Fig. 4:**
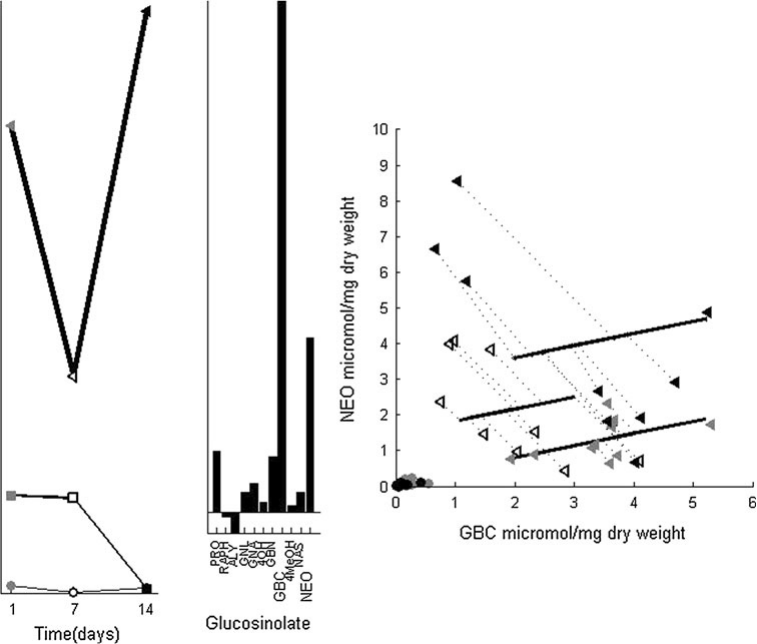
SCA-IND component 2 describing the positive relation between NEO and GBC, where the legend is identical to Fig. 3. *Left* group-level scores of component 2. The trajectory of *SJA* is given in *bold*. *Center* the loadings that indicate the positive relation between NEO and GBC. *Right* Measured NEO and GBC levels of the *SJA* plants (equal to the right panel of Fig. 3) now with the loadings and scores of component 2 superimposed. The *dotted lines* now indicate the distance between the measured and predicted NEO and GBC levels for plants harvested on all 3 days

The group-level scores of the first two SCA-IND components indicate relations between NEO and GBC after *SJA*. The first component in Fig. 3, describes the negative relation between the two. The leftmost panel of this figure shows the group-level scores of this component and the center panel the associated SCA-IND loadings. The rightmost panel shows the individual level scores and the measured data of NEO and GBC for *SJA* and control plants: in this case this is a valid representation of all chemical information in this component, as these two glucosinolates dominate its loadings. The circles and triangles show relations between the measured NEO and GBC levels for control and *SJA* plants. The levels of all *SJA* plants measured after 7 and 14 days are connected to dotted lines. These indicate the distance between the measured NEO and GBC levels in each sample and their prediction by the SCA-IND model that lies on the intersection with the continuous lines of each day. The directions of these continuous lines are the SCA-IND loadings for this component—in this figure specifically for these two glucosinolates. The dotted lines are not parallel to each other and are not orthogonal to the continuous lines that represent the loadings, which would have been the case for orthogonal projections. This shows that the results of this SCA-IND model are different from those obtained by PCA-type methods that employ this orthogonality. The length of these continuous lines, each corresponding to one harvest day, indicates the score range for that day and therefore correspond to the magnitudes of the individual differences on that day and to the group-level scores in the leftmost panel of Fig. 3. Figure 4 shows the same information for the second SCA-IND component representing the positive relation between NEO and GBC. Both figures give an insightful view on the individual differences in the way cabbage plants respond to jasmonic acid by producing NEO and GBC.

PCA components are ordered according to the amount of biochemical variation they explain, which is impossible in SCA-IND components because of the method’s mathematical properties. The biochemical background described in each component loadings is given in the center panel of both figures. Alternatively these could be represented as outer-products ${\mathbf{p}}_{r} {\mathbf{p}}_{r}^{\text{T}}$, as was done before (Jansen et al. 2011). The group-level scores are very similar to those obtained for INDSCAL described in detail before (Jansen et al. 2011). The clear-cut choice for three components in this earlier model implies that also for SCA-IND three components is appropriate; the third component will be described later. Comparison to the PCA results shows that the positive relation between both glucosinolates in the second component can be most likely attributed to the increase in NEO and GBC shared by all *SJA* plants. This means that although all plants respond to *SJA* by increasing their NEO and GBC levels, the differences in this response between individual biological replicate plants lead to larger individual differences in the levels of these glucosinolates than between control plants.

The individual-level scores also provide additional information about the individual differences: those of the first component in Fig. 3 show that the negative relation between NEO and GBC is associated with a continuous distribution of plants along the axis 7 days after *SJA*. However, 14 days after *SJA*, two distinct groups emerge along the axis, one of which has hardly-elevated GBC levels compared to control plants but considerably more NEO (e.g. sample A in Fig. 3). The other group has hardly increased NEO levels compared to control, but much more GBC—see plant B in the same figure. The positive relation between both glucosinolates is not associated with the emergence of such biological replicate groups (see rightmost panel of Fig. 4).

The first component shows that possibly two types of response emerge, although the number of plants in this study is relatively low. By the individual-level SCA-IND scores, each plant harvested 14 days after *SJA* can be identified as NEO or GBC-responder. Such subgroups of otherwise comparable biological replicates are called ‘chemotypes’ and their evolutionary reasons for existence are widely studied in chemical ecology (van Leur et al. 2006). The role of chemotype differences in the context of induced responses to herbivory are a biological concept of emerging interest (Wu et al. 2011): the SCA-IND method is tailor-made to find patterns of metabolic variation associated with such concepts.

### Root induction

**Fig. 5:**
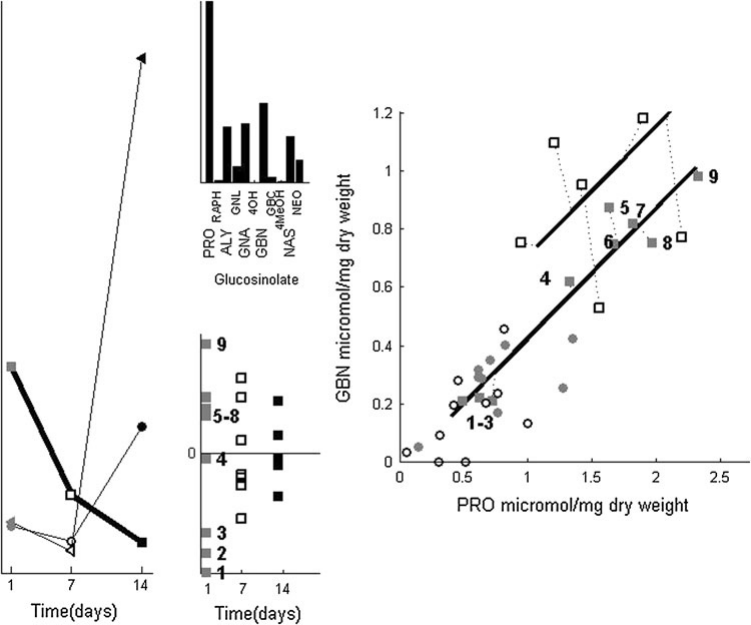
SCA-IND component 3, the legend is given in Fig. 3. *Left* group-level scores that show the early, disappearing presence of individual differences after *RJA* (trajectory given in *bold*) and the emergence late after *SJA*. *Center Top* the SCA-IND loadings of component 3. *Center bottom* Individual-level scores for component 3. that show the individual differences for the *RJA* plants. Scores of the plants harvested after 1 day are indicated by numbers. *Right* Measured PRO and GBN levels of the plants harvested 1 and 7 days after *RJA* with the loadings and scores of component 3 superimposed (analogous to the right panel of Fig. 3). The *dotted lines* indicate the difference between the measured PRO and GBN levels and their values predicted by component 3 of the SCA-IND model

Root induction leads to changes that are different from those after shoot induction. The SCA-IND model shows the individual differences in NEO and GBC levels are larger for *RJA* than for control plants until after 7 days (Fig. 4), and that the negative relation between both glucosinolates is absent (Fig. 3). However, the response to *RJA* involves individual differences in PRO, GBN and several other glucosinolates. Figure 5 shows these differences in the third SCA-IND component—the large increase 14 days after *SJA* is treated in the supplementary material. These individual differences increase already 1 day after *RJA*, where the PCA model shows increased levels of these glucosinolates only after 7 days. This implies emerging individual differences may precede consistent level changes in all individuals. The individual differences may therefore provide valuable clues to the metabolic dynamics of induced response.

The individual-level scores (Fig. 5, lower center panel) show that 1 day after *RJA*, plants 1–3 have clearly lower levels of PRO and GBN than plants 5–9. One day after *RJA* only the latter group of plants responded to *RJA*, confirmed by the measured PRO and GBN levels (Fig. 5, right). These glucosinolate levels are comparable to control plants for plants 1–3. The individual differences 7 days after *RJA* are much lower than after 1 day, as the group-level scores in Fig. 5 show. The grouping has therefore disappeared and together with the increased PCA scores (Fig. 2) this shows that all plants harvested 7 days after *RJA* have responded by increasing their PRO and GBN levels. The response time of plants to *RJA* for these glucosinolates therefore lies between 0 and 7 days.

The induced plant response, even when ‘only’ measured in 11 different but related metabolites gives rise to a series of relevant biological concepts. Involving the glucosinolate levels and their individual differences in control plants in the interpretation of response dynamics from the SCA-IND model leads to the putative distinction between early and late responders in Fig. 5. Further involvement of the a priori biochemical relation between NEO and GBC allowed the distinction of response chemotypes. The number of biological replicates in this dataset proved too low to quantify the confidence in the observed changes in individual differences (and BMRs): the jackknife approach described before (Jansen et al. 2011) lead to convergence problems. However, since also the PCA model and the raw data show the—very large—individual difference and BMR changes, the model results are reliable. The data analysis techniques already in use for metabolomics do not focus on the individual differences related to the BMRs and therefore SCA-IND gives a complementary, extremely insightful view on metabolism.

### Individual differences vs. group differences

The new view on metabolism that SCA-IND (and the individual differences) bring, turn metabolic heterogeneity—generally considered a major weakness in data analysis of biological experiments—into an invaluable information source. The most widely used methods for clustering (e.g., with PCA) and discriminant analysis aim for consistent responses between all individuals, responses in only a few of the biological replicates within the experimental group are generally disregarded. The response in PRO and GBN 1 day after *RJA* for example is not obvious in the PCA model (Fig. 2, PC 3) because these individual differences are embedded with the much larger responses in these glucosinolates that occur later for *RJA* and *SJA* plants. However, we showed here that SCA-IND can highlight these individual differences in the group and individual-levels (Fig. 5) and together with the responses shared by all individuals (e.g., described by PCA) can be used to further understanding of the metabolic behavior of biological systems.

## Conclusions

Individual differences are an innovative and complementary source of information that can be harvested to observe and interpret the biochemistry of metabolism. Such differences employ the natural variability that is inherently present as an evolutionary-driven pattern in all biological systems and complement consistent differences shared by all biological replicates. The SCA with individual differences scaling constraints (SCA-IND) models such individual differences. It combines the view on biological replicates of PCA with the BMRs that are targeted by INDSCAL.

The SCA-IND model of the metabolic response of cabbage plants to herbivory, revealed a negative relation between the levels of NEO and GBC that indicated two ‘response chemotypes’ to shoot induction, which is a concept of emerging interest. The method also revealed early and late responders to root induction, which makes SCA-IND highly fit to study dynamics with metabolomics. The SCA-IND model thereby provides insight in the chemical ecology of cabbage plants that was hitherto out-of-reach.

Individual differences are, however, of specific interest in many other fields, such as personalized nutrition and medicine. Metabolomics technology may therefore be brought to the point of direct application in, e.g., theranostics (Picard and Bergeron 2002), through individual differences metabolomics and the SCA-IND method.

## Electronic supplementary material

Below is the link to the electronic supplementary material.

Supplementary material 1 (PDF 397 kb)
