# Supplementary material for: Individual differences in metabolomics: individualised responses and between-metabolite relationships
Source: Metabolomics. 2012 Mar 15;8(Suppl 1):94–104. doi: 10.1007/s11306-012-0414-8 (PMC3337417; doi:10.1007/s11306-012-0414-8)
Supplement: Supplementary file 1 — Supplementary material 1 (PDF 397 kb) [file 11306_2012_414_MOESM1_ESM.pdf]

Supplementary Table 1 Numbers of replicates in every experimental group (combination between time after harvest and jasmonic acid treatment)

	Control	*RJA*	*SJA*
Day 1	10	9	10
Day 7	10	7	9
Day 14	10	6	9

Individual differences in PRO and GBN 14 days after *SJA*

The group-level scores in Figure 5 also show a large increase in individual differences between 7 and 14 days after for the PRO and GBN levels. Projection of these results on the glucosinolate levels (analogous to the rightmost panels of Figure 1 and Figure 5) in Supplementary Figure 1 shows that several *SJA* plants still respond this late after the treatment, which clearly illustrates the chemical and dynamic richness of the induced plant response to herbivory.

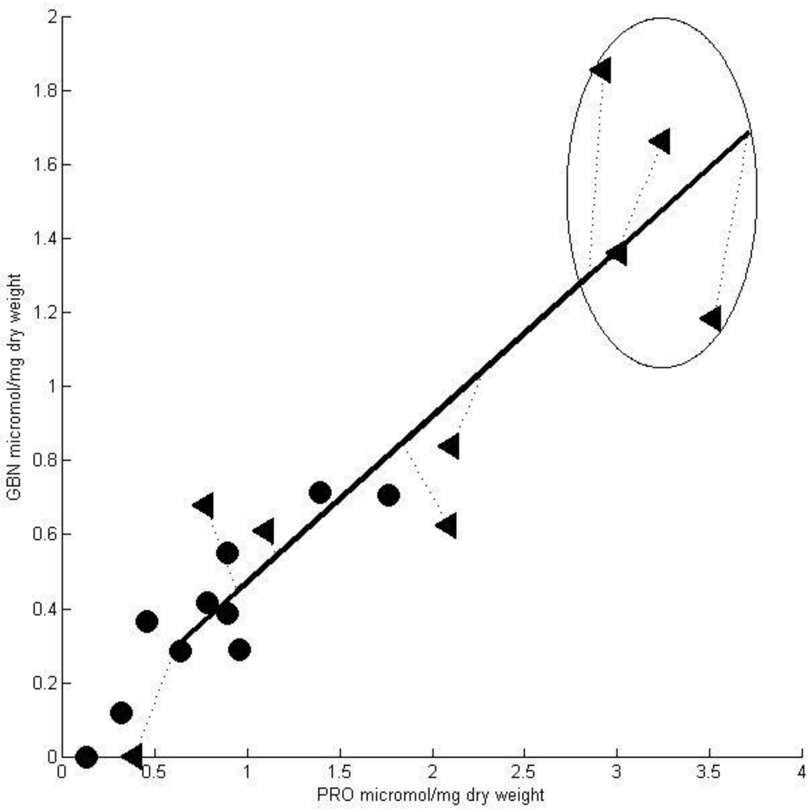

Supplementary Figure 1 Glucosinolate levels for PRO and GBN, 14 days after *SJA* (triangles) and the corresponding control plants (circles). Four out of these 9 *SJA* plants have much larger levels of these glucosinolates than control plants.
